# UBAC1/KPC2 Regulates TLR3 Signaling in Human Keratinocytes through Functional Interaction with the CARD14/CARMA2sh-TANK Complex

**DOI:** 10.3390/ijms21249365

**Published:** 2020-12-09

**Authors:** Pellegrino Mazzone, Michele Congestrì, Ivan Scudiero, Immacolata Polvere, Serena Voccola, Lucrezia Zerillo, Gianluca Telesio, Pasquale Vito, Romania Stilo, Tiziana Zotti

**Affiliations:** 1 Biogem Consortium, Via Camporeale, 83031 Ariano Irpino (AV), Italy; pellegrino.mazzone@biogem.it (P.M.); scudieroi@gmail.com (I.S.); luca.telesio@libero.it (G.T.); 2 Dipartimento di Scienze e Tecnologie, Università degli Studi del Sannio, Via Port’Arsa 11, 82100 Benevento, Italy; michele.congestri92@gmail.com (M.C.); immapolvere88@gmail.com (I.P.); romstilo@unisannio.it (R.S.); titz.zotti@gmail.com (T.Z.); 3 Genus Biotech, Università degli Studi del Sannio, Via Appia snc, 82030 Apollosa (BN), Italy; serenavoccola@gmail.com (S.V.); lucrezia.z@virgilio.it (L.Z.)

**Keywords:** CARD14, CARMA2*sh*, UBAC1, NF-κB, psoriasis, TANK, Ubiquitin, BCL10

## Abstract

CARD14/CARMA2 is a scaffold molecule whose genetic alterations are linked to human inherited inflammatory skin disorders. However, the mechanisms through which CARD14/CARMA2 controls innate immune response and chronic inflammation are not well understood. By means of a yeast two-hybrid screening, we identified the UBA Domain Containing 1 (UBAC1), the non-catalytic subunit of the E3 ubiquitin-protein ligase KPC complex, as an interactor of CARMA2*sh*, the CARD14/CARMA2 isoform mainly expressed in human keratinocytes. UBAC1 participates in the CARMA2*sh*/TANK complex and promotes K63-linked ubiquitination of TANK. In human keratinocytes, UBAC1 negatively regulates the NF-κF-activating capacity of CARMA2*sh* following exposure to poly (I:C), an agonist of Toll-like Receptor 3. Overall, our data indicate that UBAC1 participates in the inflammatory signal transduction pathways involving CARMA2*sh*.

## 1. Introduction

In human keratinocytes, the scaffold protein CARD14/CARMA2*sh* (CARMA2*sh*) controls signal transduction pathways converging on the activation of the transcription factor NF-κB, which, in turn, regulates the expression of inducible genes involved in inflammatory, proliferative and cellular stress responses [1,2]. As a consequence of resulting dis-regulated NF-κB activity, mutations in CARMA2 have been shown to be causative of several inflammatory disorders of the human skin, including familial psoriasis [2,3,4,5,6,7]. The NF-κB-inducing capacity of CARMA2*sh* relies in the assembly of a molecular complex that, in addition to CARMA2*sh*, requires the adapter molecule BCL10 and the protease MALT1 (CBM complex) [8,9]. Organization of the CBM complex by CARMA proteins occurs through the formation of higher order structures consisting of branched BCL10 filaments sheathed with MALT1 [10,11]. BCL10 filaments allow MALT1 oligomerization and activation, which eventually ensues in recruitment of the IKK complex and NF-κB activation [1,2,3,4,12]. In addition to the component of the CBM complex, and similarly to other CARMA proteins, CARMA2*sh* function is modulated by a number of CARMA2*sh*-interacting proteins [13,14,15] and by the de-ubiquitinase A20 [8]. We have also recently shown that *CARMA2sh* and the adapter protein TANK are required to activate the NF-κB response following exposure to poly (I:C), an agonist of Toll-like Receptor 3 [16]. Specifically, these data indicated that the complex CARMA2*sh*/TANK regulates the balance between inflammatory and antiviral responses in keratinocytes [16], a regulatory function shared also by the similar protein CARMA3 [17,18].

Studying molecules capable of modulating the function of CARMA2*sh* is particularly important, as it helps our understanding of the regulation of inflammatory mechanisms in human keratinocytes and they represent potential therapeutic targets for the treatment of inflammatory skin diseases [19].

Here we find that the UBA Domain Containing 1 (UBAC1), the non-catalytic subunit of the E3 ubiquitin-protein ligase KPC complex [20,21], binds to the CARMA2*sh*/TANK complex and modulates its NF-κB-inducing activity by modifying the ubiquitination state of TANK.

## 2. Results and Discussion

In order to identify novel interactors of CARMA2*sh*, we performed a panel of yeast two-hybrid assay screenings using as bait CARMA2*sh* fused to the DNA binding domain of transcription factor GAL4. A plasmid library of fusion between the transcription activation domain of GAL4 and cDNAs from human peripheral blood leucocytes was screened for interaction with GAL4-CARMA2*sh* in the yeast reporter strain AH109. Several clones that activated the β-galactosidase reporter gene were isolated. Sequence analysis revealed that several isolated plasmids, identified recurrently in multiple independent copies, encoded for the region encompassing amino acids 99-404 of UBAC1/KPC2 (UBAC1), the non-catalytic subunit of the E3 ubiquitin-protein ligase KPC complex [20,21]. Interaction of UBAC1 with CARMA2*sh* was confirmed by individual plasmid transformations in the yeast reporter strain AH109 (Table 1). 

**Figure 1:**
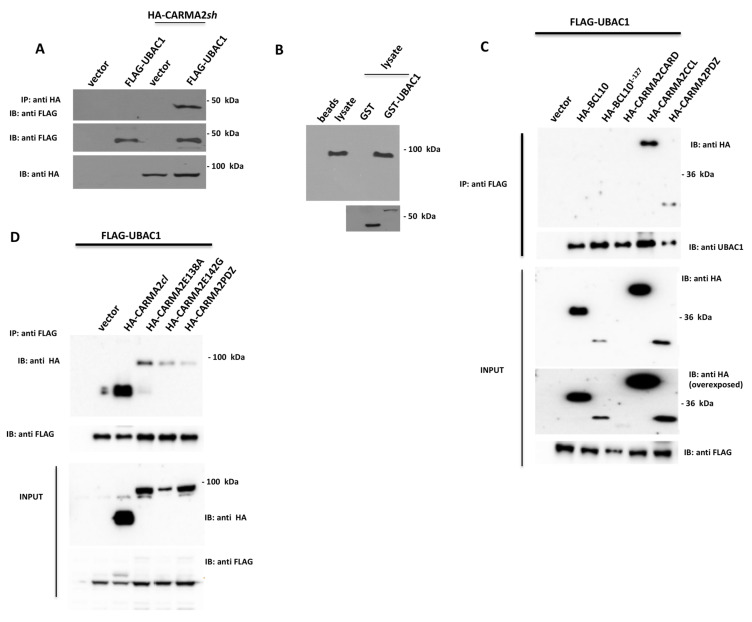
UBAC1 associates to CARMA2*sh*. (**A**) HEK293T cells were co-transfected with a plasmid encoding for FLAG-tagged UBAC1 together with an HA-tagged expression vector empty or encoding for CARMA2*sh*. A total of 24 h later, lysates were immunoprecipitated with anti-HA antibodies and analyzed for co-precipitating FLAG-UBAC1 by western blot assay. Data shown are representative of three independent experiments. (**B**) Affinity purified GST-UBAC1 was incubated with HaCaT cell lysates and western blot analysis was carried out with anti-CARMA2 and re-probing the membrane with anti-GST antibody. Data shown are representative of three independent experiments. (**C**,**D**) HEK293T cells were co-transfected with a plasmid encoding for FLAG-tagged UBAC1 together with expression vectors encoding for the indicated HA-tagged polypeptides. A total of 24 h later, lysates were immunoprecipitated with anti-FLAG antibodies and analyzed for coprecipitating proteins by western blot assay. Data shown are representative of three independent experiments.

To verify whether this interaction also occurs in mammalian cells, HEK293T cells were co-transfected with plasmids expressing FLAG-tagged UBAC1 together with a vector encoding for HA-tagged CARMA2*sh*. Cell lysates were immunoprecipitated with anti-FLAG-coated beads and the presence of coprecipitating CARMA2*sh* was assessed by immunoblot experiments probed with anti-HA antibody. The results shown in Figure 1A indicate that CARMA2*sh* coprecipitates with UBAC1 in co-transfection experiments. We further confirmed interaction of UBAC1 with endogenous CARMA2*sh* through a pull-down assay (Figure 1B). For this, lysates from HaCaT cells were incubated with recombinant GST or recombinant GST-UBAC1 fusion protein. CARMA2*sh* pull-down from HaCaT lysates was detectable using GST-UBAC1 but not GST alone, indicating that UBAC1 directly binds CARMA2*sh* in vitro. Given the modular architecture of CARMA2*sh*, we sought to determine which domain of CARMA2*sh* is involved in interaction with UBAC1. For this, CARMA2*sh* deletion mutants where co-transfected with UBAC1 and tested for interaction. As shown in Figure 1C, the polypeptides encoding for the coiled coil (CARMA2CCL, corresponding to CARMA2*sh*^123–550^) or the PDZ domain (CARMA2PDZ, corresponding to CARMA2*sh*^550–690^) of CARMA2*sh* are both able to associate with UBAC1, whereas the CARD domain (CARMA2CARD, corresponding to CARMA2*sh*^1–123^) did not. The psoriasis-linked CARMA2*sh* mutants, CARMA2*sh*E138A and CARMA2*sh*E142G, and the alternatively spliced isoform CARMA2*cl* [8] also bind to UBAC1 (Figure 1D).

**Figure 2:**
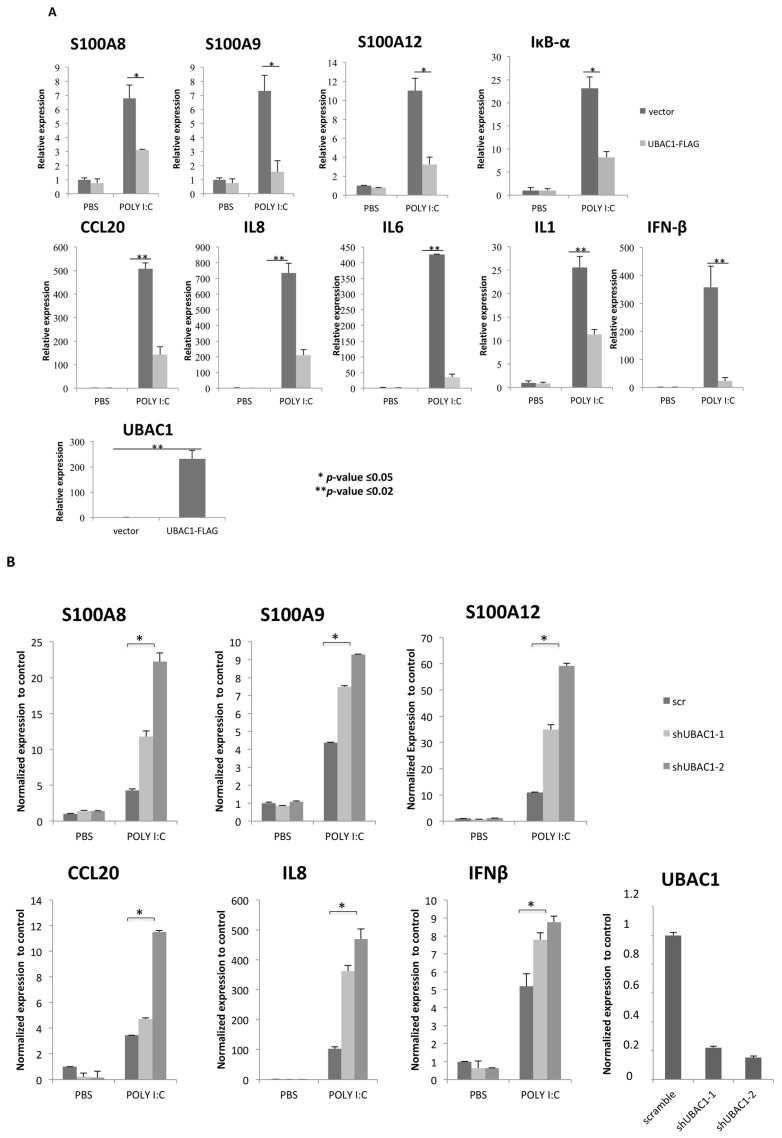
UBAC1 represses TLR3 signaling. (**A**) NHEK cells were infected with a lentiviral vector expressing UBAC1 or control GFP. A total of 48 h later, cells were left in PBS or exposed poly (I:C) (20 μg/mL) for 24 h, and the expression level of selected NF-κB and IRF3 target genes was monitored by real time PCR. Graphs show the fold-change with respect to the cells left in PBS. Data shown are representative of five independent experiments done in triplicate. Data were analyzed by Student’s *t*-test (* *p*-value ≤ 0.05; ** *p*-value ≤ 0.02). (**B**) NHEK cells were infected with retroviral vectors encoding for two different shRNAs targeting UBAC1 or a control shRNA (scramble). After selection, cells were left in PBS or exposed to poly (I:C) (20 μg/mL) for 24 h, and the expression level of selected NF-κB target genes were quantified by real-time PCR.

We have previously shown that in human keratinocytes CARMA2*sh* plays a key role in the signal transduction pathway that connects TLR3 and other pathogen-associated molecular patterns (PAMPs) recognition to NF-κB activation. Thus, in order to gain insights into the biological significance of UBAC1 in the signal transduction pathways involving CARMA2*sh*, Normal Human Epidermal Keratinocytes (NHEK) were infected with a retroviral vector encoding for UBAC1 or GFP, and the expression level of target genes such as antimicrobic peptides (S100A8, S100A9, S100A12), inflammatory cytokines (IL1, IL6, IL8, CCL20) and the IRF3-responsive anti-viral cytokine IFN-β was analyzed after exposure for 24 h to the TLR3 agonist Poly-inosinic-cytidylic acid [poly (I:C)]. Real time-PCR analysis indicates that expression of UBAC1 significantly impairs induction of TLR3 downstream target genes, acting on both NF-κB and IRF3 signaling (Figure 2A). Conversely, abrogation of UBAC1 expression in NHEK using short hairpin RNAs targeting human UBAC1 exacerbates NHEK response to TLR3 engagement (Figure 2B). 

**Figure 3:**
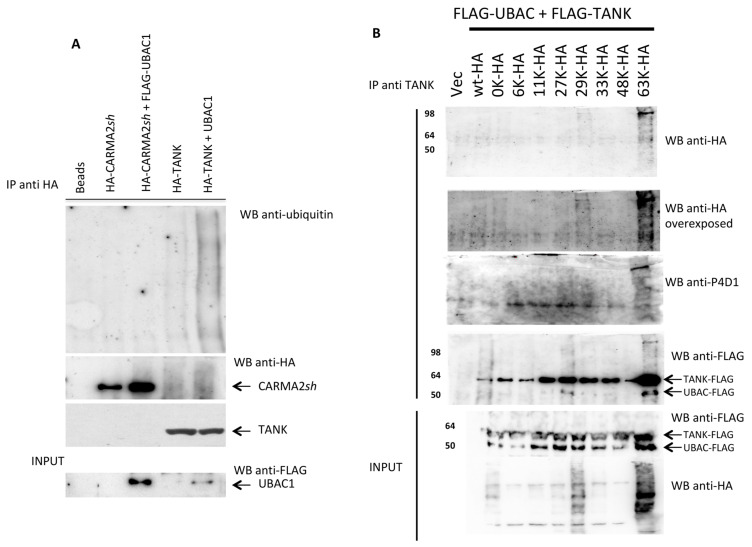
UBAC1 promotes K63-linked ubiquitination of TANK. (**A**) HEK293 cells were transfected with the indicated expression vectors. A total of 24 h later, cell lysates were immunoprecipitated with anti-HA antibody, separated by SDS-PAGE and transferred onto membranes subsequently probed with anti-ubiquitin. Statistical analysis for the blot shown in provided as supplementary Figure S1. (**B**) HEK293 cells were co-transfected with HA-tagged ubiquitin mutants and FLAG-tagged UBAC1 and TANK. The number indicates the only lysine residue remaining in the ubiquitin molecule. Immunoprecipitates with anti-TANK antibody were resolved by SDS-PAGE and blotted onto a membrane subsequently probed with anti-HA or anti- ubiquitin (P4D1). Statistical analysis for the blot shown in provided as supplementary Figure S1.

Since UBAC1 is involved in ubiquitination pathways, we verified whether UBAC1 expression modifies the ubiquitination state of CARMA2*sh* or TANK. For this, HEK293T cells were co-transfected with CARMA2*sh* or TANK in combination with UBAC1, and the ubiquitination state of these proteins was monitored by immunoblot experiments. As shown in Figure 3A, UBAC1 associates with TANK and significantly increases its ubiquitination. Because ubiquitin possesses seven lysines, and ubiquitin moieties can be conjugated through one of their lysine residues (K6, K11, K27, K29, K33, K48 and K63) or the N-terminal methionine residue (M1) [22], we used ubiquitin mutants possessing single lysine residues to investigate the nature of UBAC1-promoted polyubiquitination of TANK. 

These experiments, shown in Figure 3B, indicate that UBAC1 mostly promotes K63- and K-29-linked ubiquitination of TANK. Notably, K63- and K-29-linked ubiquitinated forms of TANK coprecipitate with UBAC1.

**Figure 4:**
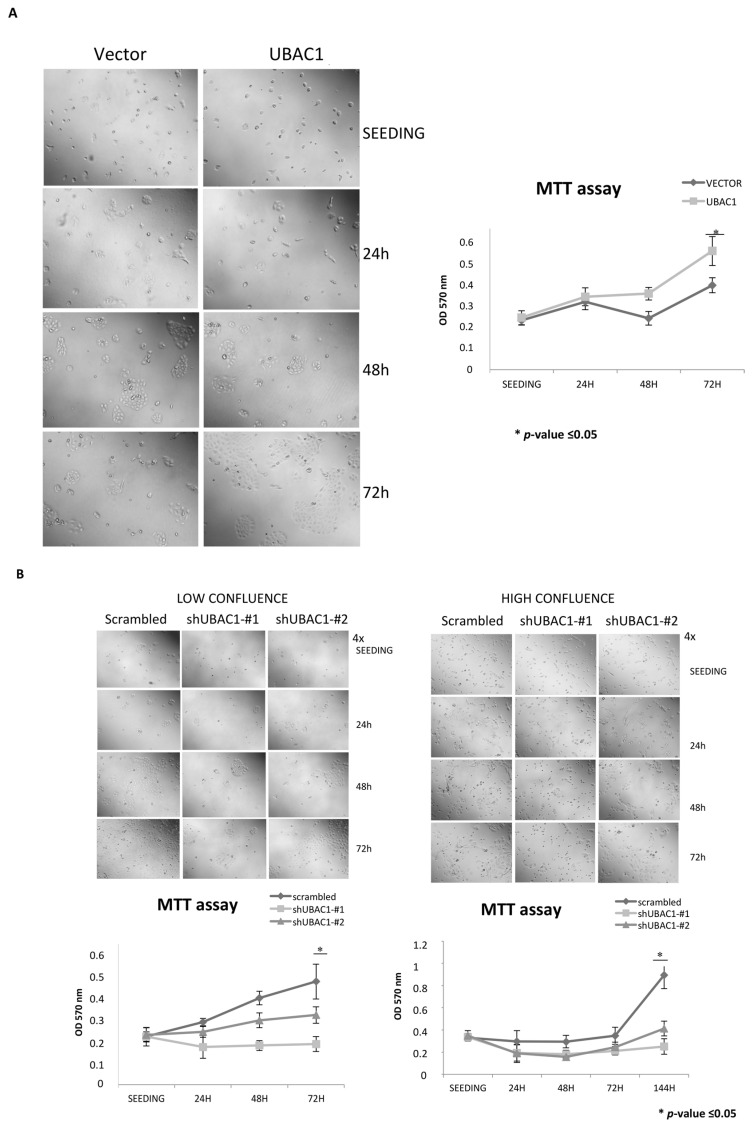
UBAC1 affects viability and proliferation in primary keratinocytes. (**A**) NHEK cells were infected with a lentiviral vector empty or expressing UBAC1. A total of 48 h later, cells were trypsinized, counted in a Neubauer chamber, and 10^4^ cells were seeded in each well of a 96-multi well plate. Cell viability was determined by MTT assay at seeding (right after cell attached to the bottom of the well) and 24, 48 and 72 h later. Representative phase contrast micrographs of transfected NHEK are also shown (magnification 4×). Graphs show the relative Optical Density at 570 nm at different time points. Data shown are representative of five independent experiments done in triplicate. Data were analyzed by Student’s t-test (* *p*-value ≤ 0.05). (**B**) NHEK cells were infected with a lentiviral vector expressing UBAC1-silencing shRNAs or scramble. A total of 48 h later, cells were trypsinized, counted with Neubauer chamber, and 10^4^ (lower confluence) or 3 × 10^4^ (higher confluence) were seeded in each well of a 96-multi well plate. MTT data and micrographs and statistical analysis were performed as in Figure 4A. When seeded at higher confluence, differences between UBAC1-silenced NHEK and control cells are evident only 144 h after seeding (micrographs not shown).

Because viability and proliferation of keratinocytes are critically regulated to maintain skin homeostasis [23], we performed an MTT assay on NHEK primary cells in which UBAC1 was ectopically expressed at seeding and 24, 48, and 72 h after. Interestingly, UBAC1 over-expression accelerates proliferation and cluster formation of NHEK cells observed at 48 and 72 h after seeding (Figure 4A). Conversely, UBAC1-silenced primary keratinocytes grow slower than controls, independently of confluence at seeding (Figure 4B). 

Genetic variations in the *CARMA2* gene are associated with familiar inflammatory skin diseases [1,2,3,4,5,6,7]. Recent studies performed using genetically modified murine strains expression CARMA2 variants associated to familiar psoriasis have shown that keratinocyte-specific expression of altered forms of CARMA2 is sufficient to trigger skin inflammation, mostly mediated by the cytokines IL-17A, IL-23 and TNFα [24,25,26,27]. However, the molecular mechanisms by which CARMA2*sh* controls inflammatory pathways are still poorly understood. We have previously shown that in human keratinocytes both the adapter protein TANK and CARMA2*sh* form a complex participating in a signal transduction pathway controlling the NF-κB response following exposure to poly I:C [16], a mimetic of double-stranded RNA and known to interact with TLR3 [28,29]. TANK has a broad role in regulating the antiviral and inflammatory immune response, promoting activation of the IRF3/IRF7 pathway as well as induction of NF-κB [30,31,32]. Here we show that UBAC1, the noncatalytic subunit of the E3 complex KPC contributes to the CARMA2*sh*/TANK complex and promotes K63-linked ubiquitination of TANK. UBAC1 was originally identified as a component of the E3 complex KPC, which includes the catalytic subunit KPC1 and KPC2/UBAC1 [20]. KPC interacts with and ubiquitinates p27 (Kip1), thereby promoting its degradation [20]. However, protein degradation by the proteosomal machinery is generally mediated by K48-linked ubiquitination of target proteins [33]. Our experiments show that UBAC1 also regulates K63-linked ubiquitination reactions by promoting K63-linked ubiquitination of TANK. Indeed, emerging evidence indicate that K63-linked ubiquitination plays a pivotal role in the regulation of pathways implicated in the inflammatory response [34,35]. Overall, our data indicate that UBAC1 participates to TLR3 signaling in keratinocytes, affecting also cell viability and proliferation. Moreover, the ubiquitination events involving UBAC1 and TANK should be considered within the molecular mechanisms that modulate the physiological function of CARMA2*sh* and of its psoriasis-linked mutants. Future work will further address this aspect.

## 3. Materials and Methods 

The two-hybrid screening. The two-hybrid screening carried on using CARMA2*sh* as bait has been described in [13]. Briefly, yeast strain AH109 GAL4^−/−^ was first transformed with pGBKT7 plasmid carrying a CARMA2*sh* cDNA bait fused with DBD of GAL4 using the lithium acetate/PEG 3000 procedure. Transformant colonies were selected by their ability to grow on a selective medium lacking tryptophan. Expression of bait GAL4DBD-CARMA2*sh* was assessed by western blot analysis. For library screening, yeast AH109 expressing GAL4DBD-CARMA2*sh* was transformed with a human peripheral blood leucocyte cDNA library cloned in pACT2 vector (Clontech, Mountain View, California, USA) in fusion with GAL4TAD. A total of 2 × 10^6^ clones were screened for interaction with GAL4DBD-CARMA2*sh* using selective growth on minimal medium lacking nutrients whose biosynthesis is mediated by genes under control of GAL4 transcriptional activity. 

Cell Culture and Tranfection. HEK293T and HaCaT cells were purchased from ATCC and cultured in Dulbecco’s modified Eagle’s medium supplemented with 10% FBS. Normal Human Epidermal Keratinocytes (NHEK) were purchased from Lonza and cultured according to the provided instructions. HEK293T were transfected using calcium phosphate precipitation; NHEK transfection was performed using DreamFect Gold Transfection Reagent (OZ Biosciences, Marseille, France) according to the manufacturer’s instruction. Retroviral infections were carried out as previously described [13]. 

GST pull-down. UBAC1-glutathione S-transferase (GST) fusion protein was produced in Escherichia coli DH5α-cells using pGEX-5x-1 as expression plasmid (GE Healthcare, Chicago, IL, USA). Transformed bacteria were grown at 37 °C until an A600 of 0.6 and induced with 1 mM isopropyl1-thio-β-D-galactopyranoside for 3 h at 37 °C. UBAC1-GST fusion protein expression was assessed by SDS-PAGE. Cells were collected in PBS and lysed with five freeze-thaw cycles followed by 1 min of sonication. After centrifugation, the soluble fraction was bound to glutathione-Sepharose beads (GE Healthcare, Chicago, IL, USA) overnight at 4 °C in an EconoPac chromatography column (Bio-Rad) on a rotating wheel. Beads were then washed three times with PBS and resuspended in Lysis Buffer with protease inhibitors (Roche Applied Science). Lysates obtained from HaCaT cells were mixed with Sepharose-bound GST-UBAC1 (1.5 μg) overnight at 4 °C on a rotating wheel. Beads were centrifugated, washed three times with lysis buffer without protease inhibitors, and resuspended in reducing Sample Buffer. Samples were boiled for 5 min, and supernatants were separated by SDS-PAGE followed by blotting and incubation with CARMA2 antibody.

Immunoblot analysis and coprecipitation. Cell lysates were made in lysis buffer (150 mM NaCl, 20 mM Hepes, pH 7.4, 1% Triton X-100, 10% glycerol) and a mixture of proteases inhibitors (Roche, Basel, Switzerland) according to the manufacturer’s instructions. Proteins were separated by SDS–PAGE, transferred onto nitrocellulose membrane, and incubated with primary antibodies followed by horseradish peroxidase-conjugated secondary antibodies (Amersham Biosciences, Little Chalfont, UK). Blots were developed using the ECL system (Amersham Biosciences, Little Chalfont, UK). For co-immunoprecipitation experiments, cells were lysed in lysis buffer and immunocomplexes were bound to protein A/G (Amersham Biosciences, Little Chalfont, UK) for 2 h at 4 °C. Immunocomplexes were extensively washed, resolved by SDS–PAGE, and analyzed by immunoblot assay. Sources of antisera and monoclonal antibodies were the following—anti-FLAG, anti-β-Actin, Sigma; anti-HA, anti-TANK anti-CARMA2, anti-ubiquitin (P4D1), Santa Cruz Biotechnology. 

Real-time RT-PCR. Total RNA was isolated from cells using TRIzol reagent (Invitrogen, Carlsbad, CA, USA). The reverse transcriptase reaction was performed using 1 μg of total RNA in a 20 μL reaction. A total of 10 ng of the resulting cDNA was used in the subsequent amplification step along with 300 nM of each primer. For the data analysis, the geometric mean values of β−actin and succinyl-CoA synthetase β-subunit fragment were used for the normalization. The ΔΔCt method was used for the relative transcription level calculation. Real-time PCR reactions were performed in triplicate by using the SYBR Green PCR Master Mix (Qiagen, Hilden, Germany) in a 7900HT system (Applied Biosystems, Foster City, CA, USA). shRNAs targeting UBAC1 were obtained from Sigma-Aldrich (St. Louis, MO, USA) and have the following sequence: 

TRCN0000074948 (shRNA1)

CCGGGCTGTGGTAATTCTATTTGTACTCGAGTACAAATAGAATTACCACAGCTTTTTG

TRCN0000291039 (shRNA2)

CCGGCGGACTATTGTTCAGCTAGAACTCGAGTTCTAGCTGAACAATAGTCCGTTTTTG

For cell treatment, after attaining confluence, the culture medium of the NHEK cell monolayers was removed. Fresh medium with 20 μg/mL Poly (I:C) was added to cells for 24 h at 37 °C, 95% humidity and 5% CO_2_. At the end of incubation, cells were washed with cell culture medium and lysed with TRI Reagent (Sigma-Aldrich, St. Louis, MO, USA). Lysed cells were collected and stored at −80 °C until real-time time polymerase chain reaction (PCR) was performed.

MTT assay. At the end of each testing time, MTT solution (0.5 mg/mL) was added to each well, and the plates were incubated for 1 h at 37 °C. The MTT solution was removed, and isopropyl alcohol was added in order to dissolve formazan crystals. The absorbance at 570 nm was read on a microplate spectrophotometer (Applied Biosystem, Foster City, CA, USA) and background absorbance was subtracted to raw optical densities. Graphs are representative of three replicates and error bars are calculated as standard deviations. 

## Figures and Tables

**Table 1:** Interaction of CARMA2sh with UBAC1 in the yeast two-hybrid assay. Yeast AH109 was transformed with CARMA2sh fused to the GAL4-DNA binding domain together with the indicated cDNAs fused to GAL4-activating domain. The cDNA encoding for FADD was used as a putative negative control. Interactions were examined by yeast growth on selective media; assays were done for ten independent transformants. Yeast colonies were scored as positive (+) when a growth developed within 36 h; a negative (-) was scored when growth failed to develop within 5 days.

Protein Fused to GAL4 Domain		Yeast Growth on Selective Media
DNA-Binding	Activating	
-	UBAC1	-
Vector	UBAC1	-
FADD	UBAC1	-
CARMA2*sh*	UBAC1	+++
CARMA2*sh*	-	-

## Supplementary Materials

Supplementary materials can be found at https://www.mdpi.com/1422-0067/21/24/9365/s1.
