# Supplementary material for: UBAC1/KPC2 Regulates TLR3 Signaling in Human Keratinocytes through Functional Interaction with the CARD14/CARMA2sh-TANK Complex
Source: Int J Mol Sci. 2020 Dec 9;21(24):9365. doi: 10.3390/ijms21249365 (PMC7764236; doi:10.3390/ijms21249365)
Supplement: Supplementary file 1 [file ijms-21-09365-s001.pdf]

International Journal of
Molecular Sciences

MDPI

Supplementary. Material.

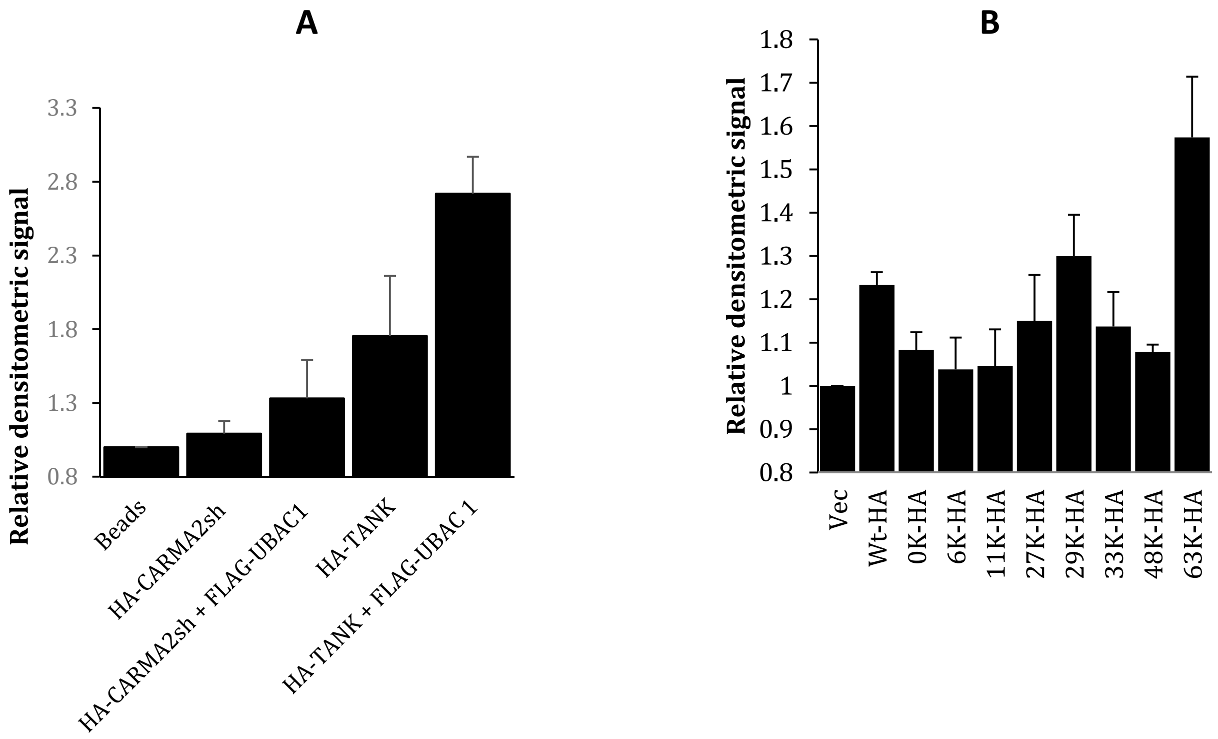

Figure S1. Densitometric analysis of the blots shown in A and B. Signals from two independent experiments were acquired and analyzed with Imagej software.
